# Supplementary material for: Genome-wide identification of candidate aquaporins involved in water accumulation of pomegranate outer seed coat
Source: PeerJ. 2021 Jul 15;9:e11810. doi: 10.7717/peerj.11810 (PMC8286702; doi:10.7717/peerj.11810)
Supplement: Supplemental Information 5 [file peerj-09-11810-s005.docx]

**Figure S 2**

Conserved domain analysis of aquaporins identified in pomegranate using CDD tool from NCBI

| Query | PSSM-ID | E-Value | Bitscore | Accession | Short name | Superfamily |
| --- | --- | --- | --- | --- | --- | --- |
| *PgrNIP1.1* | 350945 | 2.13E-128 | 366.654 | cl00200 | MIP superfamily | - |
| *PgrNIP1.2* | 350945 | 3.95E-130 | 370.891 | cl00200 | MIP superfamily | - |
| *PgrNIP2.1* | 350945 | 4.89E-80 | 243.663 | cl00200 | MIP superfamily | - |
| *PgrNIP4.1* | 350945 | 3.52E-123 | 352.674 | cl00200 | MIP superfamily | - |
| *PgrNIP4.2* | 350945 | 5.29E-136 | 384.646 | cl00200 | MIP superfamily | - |
| *PgrNIP5.1* | 177663 | 2.52E-133 | 377.665 | PLN00026 | PLN00026 | cl00200 |
| *PgrNIP7.1* | 350945 | 1.01E-114 | 331.821 | cl00200 | MIP superfamily | - |
| *PgrNIP3.1* | 350945 | 5.72E-71 | 220.663 | cl00200 | MIP superfamily | - |
| *PgrTIP1.1* | 177664 | 6.50E-148 | 413.028 | PLN00027 | PLN00027 | cl00200 |
| *PgrTIP1.2* | 177664 | 5.64E-148 | 413.028 | PLN00027 | PLN00027 | cl00200 |
| *PgrTIP1.3* | 177664 | 5.53E-135 | 380.286 | PLN00027 | PLN00027 | cl00200 |
| *PgrTIP1.5* | 177664 | 2.09E-150 | 419.191 | PLN00027 | PLN00027 | cl00200 |
| *PgrTIP1.6* | 177664 | 2.14E-153 | 426.895 | PLN00027 | PLN00027 | cl00200 |
| *PgrTIP1.7* | 177664 | 3.29E-132 | 373.352 | PLN00027 | PLN00027 | cl00200 |
| *PgrTIP1.8* | 177664 | 8.26E-148 | 413.028 | PLN00027 | PLN00027 | cl00200 |
| *PgrTIP2.1* | 350945 | 1.46E-120 | 343.457 | cl00200 | MIP superfamily | - |
| *PgrTIP2.3* | 350945 | 1.57E-130 | 368.881 | cl00200 | MIP superfamily | - |
| *PgrTIP3.1* | 177664 | 1.02E-103 | 301.32 | PLN00027 | PLN00027 | cl00200 |
| *PgrTIP3.2* | 350945 | 2.19E-89 | 265.111 | cl00200 | MIP superfamily | - |
| *PgrTIP4.1* | 350945 | 1.67E-85 | 254.711 | cl00200 | MIP superfamily | - |
| *PgrTIP5.1* | 350945 | 1.28E-65 | 204.786 | cl00200 | MIP superfamily | - |
| *PgrTIP5.2* | 350945 | 8.27E-110 | 316.845 | cl00200 | MIP superfamily | - |
| *PgrPIP1.1* | 333943 | 4.03E-98 | 287.289 | pfam00230 | MIP | cl00200 |
| *PgrPIP1.3* | 333943 | 8.62E-94 | 276.118 | pfam00230 | MIP | cl00200 |
| *PgrPIP1.2* | 333943 | 1.51E-89 | 265.332 | pfam00230 | MIP | cl00200 |
| *PgrPIP1.4* | 333943 | 3.59E-87 | 259.554 | pfam00230 | MIP | cl00200 |
| *PgrPIP1.5* | 333943 | 6.53E-95 | 279.2 | pfam00230 | MIP | cl00200 |
| *PgrPIP2.1* | 333943 | 1.70E-92 | 272.651 | pfam00230 | MIP | cl00200 |
| *PgrPIP2.2* | 333943 | 4.24E-92 | 271.881 | pfam00230 | MIP | cl00200 |
| *PgrPIP2.7* | 333943 | 1.61E-97 | 285.363 | pfam00230 | MIP | cl00200 |
| *PgrPIP2.8* | 333943 | 5.18E-96 | 281.896 | pfam00230 | MIP | cl00200 |
| *PgrPIP2.5* | 333943 | 6.05E-93 | 273.807 | pfam00230 | MIP | cl00200 |
| *PgrPIP2.6* | 333943 | 1.16E-93 | 275.733 | pfam00230 | MIP | cl00200 |
| *PgrPIP2.3* | 333943 | 6.93E-92 | 271.11 | pfam00230 | MIP | cl00200 |
| *PgrPIP2.4* | 333943 | 8.51E-92 | 271.11 | pfam00230 | MIP | cl00200 |
| *PgrXIP2.1* | 350945 | 2.57E-25 | 100.852 | cl00200 | MIP superfamily | - |
| *PgrSIP1.1* | 350945 | 4.46E-14 | 68.8194 | cl00200 | MIP superfamily | - |
| *PgrSIP1.2* | 350945 | 1.21E-09 | 56.493 | cl00200 | MIP superfamily | - |
