# Supplementary material for: Genome-wide identification of candidate aquaporins involved in water accumulation of pomegranate outer seed coat
Source: PeerJ. 2021 Jul 15;9:e11810. doi: 10.7717/peerj.11810 (PMC8286702; doi:10.7717/peerj.11810)
Supplement: Supplemental Information 6 [file peerj-09-11810-s006.docx]

**Figure S2.** Transmembrane domains analysis of PgrAQP in pomegranate

|  |  |  | **TMHMM** |  |  | **SOSUI** |  |  |
| --- | --- | --- | --- | --- | --- | --- | --- | --- |
| **gene** | **Length** | **ExpAA** | **First 60** | **predHel** |  | **protein type** | **helix** | **Max TM** |
| *PgrNIP1.1* | 288 | 127.80 | 9.5 | 6 |  | MP | 6 | **6** |
| *PgrNIP1.2* | 278 | 113.3 | 16.69 | 5 |  | MP | 6 | **6** |
| *PgrNIP2.1* | 294 | 117.66 | 8.19 | 5 |  | MP | 6 | **6** |
| *PgrNIP4.1* | 278 | 130.97 | 11.56 | 6 |  | MP | 6 | **6** |
| *PgrNIP4.2* | 270 | 138.49 | 20.49 | 6 |  | MP | 6 | **6** |
| *PgrNIP5.1* | 245 | 86.72 | 0.05 | 4 |  | MP | 4 | **4** |
| *PgrNIP7.1* | 304 | 141.34 | 0.005 | 6 |  | MP | 6 | **6** |
| *PgrNIP3.1* | 280 | 123.96 | 9.59 | 5 |  | MP | 6 | **6** |
| *PgrTIP1.1* | 252 | 134.34 | 24.52 | 6 |  | MP | 6 | **6** |
| *PgrTIP1.2* | 252 | 138.84 | 24.44 | 6 |  | MP | 6 | **6** |
| *PgrTIP1.3* | 250 | 133.82 | 26.04 | 6 |  | MP | 6 | **6** |
| *PgrTIP1.5* | 251 | 134.60 | 22.67 | 6 |  | MP | 6 | **6** |
| *PgrTIP1.6* | 251 | 143.84 | 21.41 | 6 |  | MP | 7 | **7** |
| *PgrTIP1.7* | 252 | 133.51 | 25.32 | 6 |  | MP | 6 | **7** |
| *PgrTIP1.8* | 258 | 142.38 | 21.44 | 7 |  | MP | 6 | **7** |
| *PgrTIP2.1* | 248 | 153.89 | 29.20 | 7 |  | MP | 7 | **7** |
| *PgrTIP2.3* | 250 | 138.16 | 28.2 | 6 |  | MP | 6 | **6** |
| *PgrTIP3.1* | 261 | 137.5 | 22.96 | 6 |  | MP | 6 | **6** |
| *PgrTIP3.2* | 259 | 138.37 | 23.86 | 6 |  | MP | 5 | **6** |
| *PgrTIP4.1* | 247 | 151.27 | 33.76 | 7 |  | MP | 7 | **7** |
| *PgrTIP5.1* | 264 | 145.77 | 21.81 | 6 |  | MP | 5 | **6** |
| *PgrTIP5.2* | 259 | 135.61 | 26.59 | 6 |  | MP | 6 | **6** |
| *PgrPIP1.1* | 287 | 131.34 | 5.14 | 6 |  | MP | 6 | **6** |
| *PgrPIP1.3* | 287 | 126.07 | 6.86 | 6 |  | MP | 6 | **6** |
| *PgrPIP1.2* | 287 | 130.68 | 6.94 | 6 |  | MP | 6 | **6** |
| *PgrPIP1.4* | 289 | 127.55 | 5.22 | 6 |  | MP | 4 | **6** |
| *PgrPIP1.5* | 289 | 130.24 | 3.52 | 6 |  | MP | 6 | **6** |
| *PgrPIP2.1* | 281 | 135.43 | 21.67 | 6 |  | MP | 6 | **6** |
| *PgrPIP2.2* | 286 | 134.02 | 22.47 | 6 |  | MP | 5 | **6** |
| *PgrPIP2.7* | 286 | 129.48 | 20.79 | 6 |  | MP | 5 | **6** |
| *PgrPIP2.8* | 286 | 130.26 | 20.76 | 6 |  | MP | 5 | **4** |
| *PgrPIP2.5* | 285 | 126.99 | 22.36 | 5 |  | MP | 5 | **6** |
| *PgrPIP2.6* | 288 | 131.34 | 19.92 | 6 |  | MP | 5 | **6** |
| *PgrPIP2.3* | 283 | 134.36 | 20.87 | 6 |  | MP | 4 | **6** |
| *PgrPIP2.4* | 289 | 139.07 | 20.93 | 6 |  | MP | 5 | **6** |
| *PgrXIP2.1* | 317 | 123.22 | 1.23 | 6 |  | MP | 6 | **6** |
| *PgrSIP1.1* | 241 | 124.27 | 38.83 | 5 |  | MP | 5 | **5** |
| *PgrSIP1.2* | 240 | 125.97 | 39.39 | 5 |  | MP | 6 | **6** |

Exp AA: The expected number of amino acids in transmembrane helices.

First 60: The expected number of amino acids in transmembrane helices in the first 60 amino acids of the protein.

MP: Membrane protein

TMH: The number of predicted transmembrane helices.

MAX TMH: The maximum number among the TMH predicted by TMHMM and SOSUI

^#^ indicates sequences showing less than 6 transmembrane domains based on their sequence alignment.
