# Supplementary material for: Genome-wide identification of candidate aquaporins involved in water accumulation of pomegranate outer seed coat
Source: PeerJ. 2021 Jul 15;9:e11810. doi: 10.7717/peerj.11810 (PMC8286702; doi:10.7717/peerj.11810)
Supplement: Supplemental Information 7 — (A) The PgrAQPs phylogenetic tree was created by the NJ method. The five subfamilies of AQP genes were marked in orange-yellow (TIPs), blue (PIPs), pink (NIPs), red (XIP) and green (SIPs), respectively. (B) Exon-intron structure of PgrAQP genes. The boxes denote exons within coding regions, and the lines connecting them represent introns. (C) Motifs compositions of the PgrAQP proteins were identified by MEME tools. [file peerj-09-11810-s007.docx]

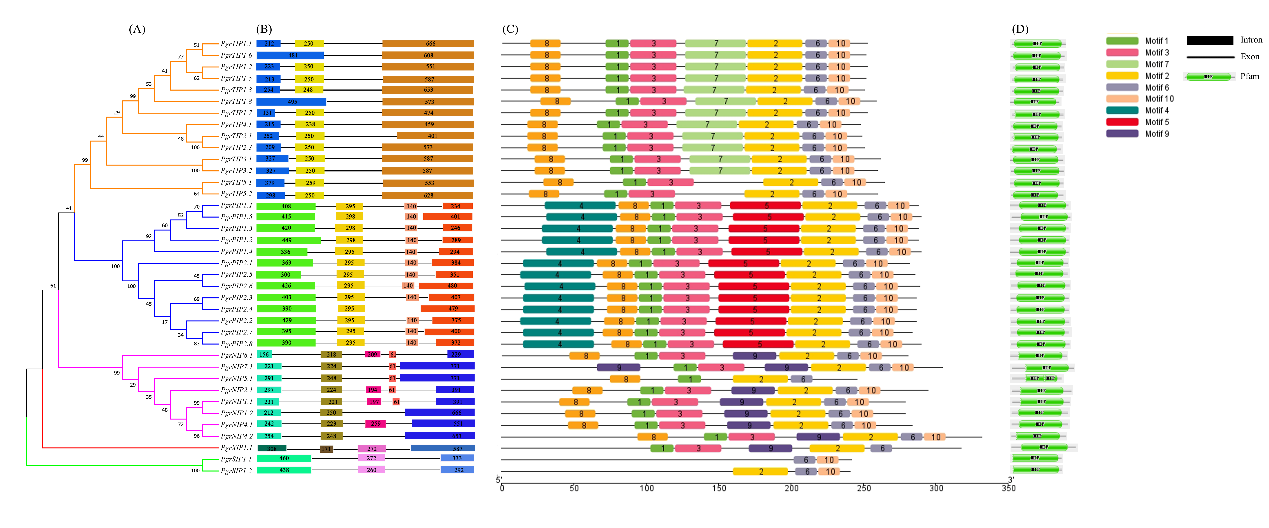


**Figure S3. Phylogenetic relationships, gene structures and conserved motifs compositions of PgrAQPs.** **(A)** The PgrAQPs phylogenetic tree was created by the NJ method. The five subfamilies of AQP genes were marked in orange-yellow (TIPs), blue (PIPs), pink (NIPs), red (XIP) and green (SIPs), respectively. **(B)** Exon-intron structure of PgrAQP genes. The boxes denote exons within coding regions, and the lines connecting them represent introns. **(C)** Motifs compositions of the PgrAQP proteins were identified by MEME tools.
